# Fractional Carbon Dioxide Laser for Keratosis Pilaris: A Single-Blind, Randomized, Comparative Study

**DOI:** 10.1155/2016/1928540

**Published:** 2016-05-09

**Authors:** Vasanop Vachiramon, Pattarin Anusaksathien, Silada Kanokrungsee, Kumutnart Chanprapaph

**Affiliations:** Division of Dermatology, Faculty of Medicine Ramathibodi Hospital, Mahidol University, Bangkok 10400, Thailand

## Abstract

*Objective*. Keratosis pilaris (KP) is a common condition which can frequently be cosmetically disturbing. Topical treatments can be used with limited efficacy. The objective of this study is to evaluate the effectiveness and safety of fractional carbon dioxide (CO_2_) laser for the treatment of KP.* Patients and Methods*. A prospective, randomized, single-blinded, intraindividual comparative study was conducted on adult patients with KP. A single session of fractional CO_2_ laser was performed to one side of arm whereas the contralateral side served as control. Patients were scheduled for follow-up at 4 and 12 weeks after treatment. Clinical improvement was graded subjectively by blinded dermatologists. Patients rated treatment satisfaction at the end of the study.* Results*. Twenty patients completed the study. All patients stated that the laser treatment improved KP lesions. At 12-week follow-up, 30% of lesions on the laser-treated side had moderate to good improvement according to physicians' global assessment (*p* = 0.02). Keratotic papules and hyperpigmentation appeared to respond better than the erythematous component. Four patients with Fitzpatrick skin type V developed transient pigmentary alteration.* Conclusions*. Fractional CO_2_ laser treatment may be offered to patients with KP. Dark-skinned patients should be treated with special caution.

## 1. Introduction

Keratosis pilaris (KP) is a common disorder of keratinization. It is characterized by multiple tiny follicular keratotic papules that may have surrounding erythema. Hyperpigmentation can sometimes occur especially in individuals with dark-complexioned skin. KP mainly involves the extensor arms, back, anterior thighs, face, and buttock. The exact prevalence is difficult to estimate but could be found up to 50% of the general population [1, 2]. Although KP has no impact on general health, its influence on the quality of life arises especially for those with lesions on the exposed areas. Treatment options include emollients, keratolytics, and topical steroids when necessary [3]. However, the result is highly variable and recurrence following treatment discontinuation is often problematic.

Over the past decade, attempts to eradicate KP through various laser and light-based therapy have been investigated. This includes 532 nm potassium titanyl phosphate laser; 595 nm pulsed dye laser; 1064 nm Q-switched Nd:YAG laser; long-pulse 1064 nm Nd:YAG laser; combination of 595 nm pulse dye laser, long-pulse 755 nm alexandrite laser, and microdermabrasion [4–9]. To the best of our knowledge, there has never been a report on the effectiveness of fractional carbon dioxide laser (CO_2_) for the treatment of KP. We hypothesize that fractional CO_2_ laser could remove the keratotic component and brown pigmentation of KP. The objective of this study is to evaluate the efficacy and safety of fractional CO_2_ laser for the treatment of KP.

## 2. Materials and Methods

This study is a prospective, randomized, single-blinded, intraindividual comparative study. The study protocol has been approved by Mahidol University Institutional Review Board for Human Subject Research (Protocol number 015821). The study confirmed the guidelines of the declaration of Helsinki. All study subjects had obtained the inform consent at the enrollment.

### 2.1. Patient

Subjects were recruited from an outpatient dermatology clinic at a university-based hospital (Ramathibodi Hospital, Mahidol University, Bangkok, Thailand). Healthy patients aged 18 years or older with the presence of KP on both sides of arms were eligible. Patients with history of keloid or hypertrophic scar and those who were pregnant or lactating were excluded from the study. Patients who received topical medications or emollients within 1 month or had performed any laser treatment or dermabrasion for KP within past 6 months were also excluded. In addition, we eliminated patients with prior history of medication affecting keratinization (e.g., isotretinoin and acitretin) within the past 3 years from this trial.

After enrollment, informed consent had been obtained and demographic data was recorded. Using the table of randomization (a table of random numbers), the left side and right side of the arms were randomly allocated to receive laser treatment. One side received fractional CO_2_ laser therapy (side A) and the other (side B) did not receive laser treatment.

### 2.2. Treatment Regimens

Fractional CO_2_ laser therapy using a 10,600 nm eCO_2_ laser (Lutronic Corporation, Goyang, Republic of Korea) was performed to the lesions on side A. This was a single session laser treatment. The settings were the pulse energy of 24–30 mJ and spot density of 300 spots/cm^2^ in static mode; 2 passes were delivered using a 300-density tip. To minimized pain, local anesthetic cream (a eutectic mixture of local anesthetics, Astra Zeneca LP, Wilmington, DE) was applied under occlusion for 30 minutes before the laser treatment and air cooling with a cold air cooling device (CRIOjet AIR Mini CRIO Medizintechnik GmbH, Birkenfeld, Germany) at a cooling level of 4 was used during the laser therapy.

**Figure 1:**
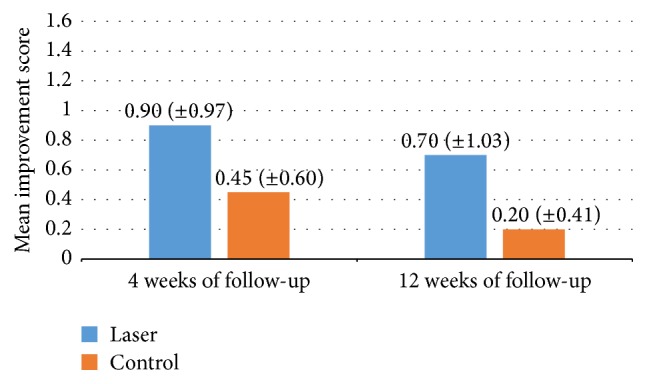
Mean improvement score at 4-week and 12-week follow-up.

After the laser treatment, petrolatum ointment was applied to the lesions on side A twice a day for 5 days. To avoid confounding effect of petrolatum ointment, the lesions on side B also received petrolatum ointment twice daily for 5 days. The patients were appointed for follow-up on the 4th and 12th week after the last treatment (Figure 1).

### 2.3. Outcome Evaluation

Standard digital photograph was taken at baseline, 4 weeks, and 12 weeks after the last treatment. Two dermatologists who did not perform the laser procedure evaluated the response through digital images. Global improvement score, keratotic papules, hyperpigmentation, and erythema were evaluated using the grading system for improvement. The grading scale is as follows: grade −4, >75% worsening; grade −3, 51–75% worsening; grade −2, 26–50% worsening; grade −1, 1–25% worsening; grade 0, no change; grade 1, 1–25% improvement (minimal); grade 2, 26–50% improvement (moderate); grade 3, 51–75% improvement (good); grade 4, >75% improvement (excellent).

Patient satisfaction was assessed at the end of study (12th week of follow-up). They were asked to rate the overall improvement and satisfaction by grading score, that is, 0, unsatisfied; 1, poor; 2, fair; 3, satisfied; 4, extremely satisfied. Pain score was also evaluated immediately after laser treatment using 10-point visual analogue scale (VAS, 0–10), in which 0 indicated no pain at all and 10 indicated extreme pain. Possible adverse events (e.g., erythema, burning sensation, hyperpigmentation, hypopigmentation, scarring, and infection) were recorded at every visit.

### 2.4. Statistical Analyses

Categorical variables were expressed as percentages. Continuous variables (e.g., physician grading score and VAS) were expressed as mean ± standard deviation or median (range). *t*-test was used to compare continuous data between two dependent samples. All analyses were performed using STATA version 13 (Stata Corp., College Station, Texas, USA). A *p* value of 0.05 or less was considered statistically significant.

## 3. Results

Twenty-four eligible patients with KP on both arms were enrolled in this study. Four patients lost to follow-up at 12 weeks after treatment for reasons unrelated to laser treatment (i.e., inconvenient follow-up schedule). Twenty patients completed the 12-week study and were included in the analysis (20 arms on side A and 20 arms on side B). Twelve were men (60%) and 8 were women (40%). The median age of subject was 27 years old (19–32). The median age of KP onset was 15 years old (10–22). Eight patients were Fitzpatrick skin type III, 5 patients were Fitzpatrick skin type IV, and 7 patients were Fitzpatrick skin type V.

### 3.1. Global Assessments

At 4 weeks after treatment, grade 2 or more improvement was achieved in 4 arms on side A and 1 arm on side B (*p* = 0.097). At 12 weeks of follow-up, 6 arms on side A achieved grade 2 or more improvement but no KP lesion on side B achieved such response (*p* = 0.02). None of the patients experienced worsening of the lesions (Table 1).

**Figure 2:**
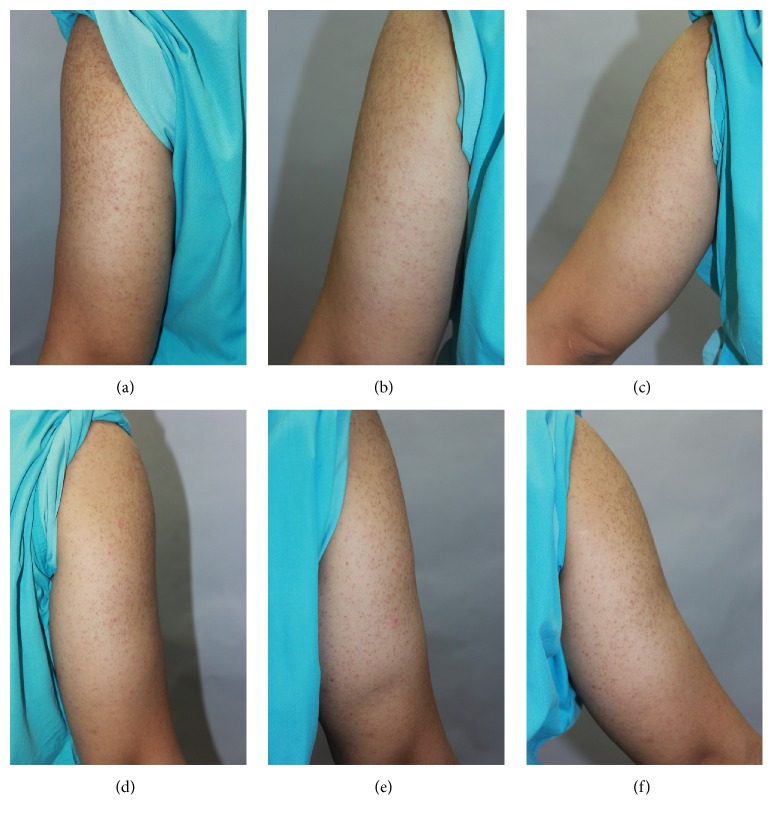
Photograph of patient before (a, d); at 4-week follow-up (b, e); at 12-week follow-up (c, f). Left arm was treated with fractional CO_2_ laser (a, b, c) and right arm was served as control (d, e, f). Left arm showed grade 3 improvement at 12-week follow-up (c).

In terms of mean improvement score, the score was 0.90 (±0.97) on side A and 0.45 (±0.60) on side B (*p* = 0.08) at 4 weeks after treatment. The mean improvement scores on side A and side B at 12 weeks of follow-up were 0.7 (±1.03) and 0.2 (±0.41), respectively (*p* = 0.05) (Figures 1 and 2). There was no statistically significant difference according to sex, age, or disease duration.

### 3.2. Keratotic Papules

The evaluators assessed overall improvement of keratotic papules but the actual number was not counted. Similar to global assessments, higher grade of improvement (grade 2 or more) was found in 4 arms on side A and 1 arm on side B at 4 weeks of follow-up. At 12 weeks of follow-up, at least grade 2 improvement was achieved in 5 arms on side A and 1 arm on side B. No study subjects experienced worsening of keratotic papules (Table 2).

### 3.3. Hyperpigmentation

Grade 2 or more improvement in hyperpigmentation was achieved in 5 patients on side A at 4 weeks of follow-up. At 12 weeks of follow-up, there were 6 arms on side A that achieved grade 2 or more improvement. Majority of lesions on side B showed no improvement in terms of pigmentation at both the 4th and 12th week of follow-up (Table 3).

### 3.4. Erythema

Grade 3 improvement in erythema was achieved on 2 arms of side A at 4 weeks of follow-up but no lesions on side B achieved similar response. At 12 weeks, grade 2 and grade 3 improvement were found in 2 patients each on side A. None of KP lesions on side B achieved grade 2, 3, or 4 improvement at 12 weeks. No patients had worsening of erythema after laser.

### 3.5. Patient Satisfaction

All patients rated the lesions as improved on side A. Improvement of seven lesions on side A (35%) was marked as satisfied or extremely satisfied whereas no lesion on side B was rated as satisfied or extremely satisfied (*p* = 0.08) (Table 4).

### 3.6. Adverse Events

**Figure 3:**
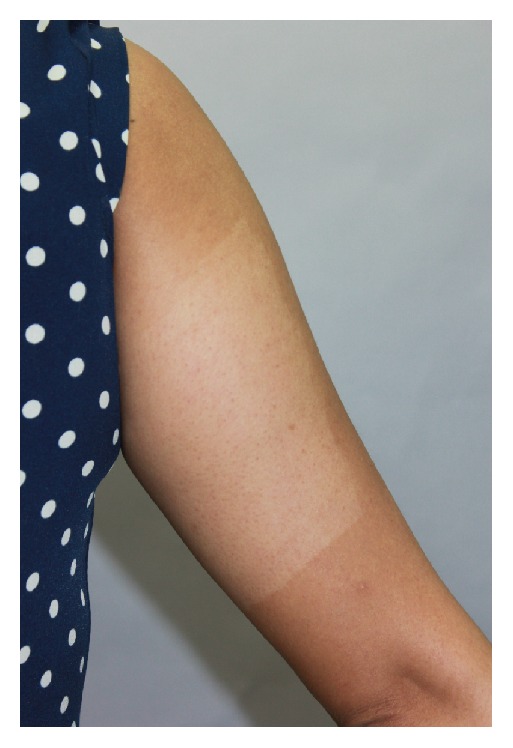
Hypopigmentation occurred on the laser-treated side at 4-week follow-up.

Pain was observed on the laser-treated side in all patients. The mean pain score was 4.2 (±2.6). Erythema and burning sensation were also observed in all laser-treated lesions. The median duration of erythema was 2 days (1–5) and median duration of burning sensation was 24 hours (1–72). Two patients experienced hyperpigmentation on laser-treated side at 4 weeks of follow-up, both of which had spontaneous improvement at 12 weeks after treatment. Hypopigmentation developed on 2 lesions of side A: the first lesion spontaneously improved at 4-week follow-up and another lesion improved without any treatment at 12-week follow-up (Figure 3). No infection or scarring was observed. All patients who developed hyperpigmentation or hypopigmentation were Fitzpatrick skin type V.

## 4. Discussion

KP is a very common condition among the general population but few treatment options with promising and sustained results exist. Although it is often asymptomatic, it can cause pruritus in some patients. In addition, the affected skin resembling gooseflesh resulting in unsightly appearance may lead to psychological distress. Treatment options are variable due to various aspects of the lesions. In most cases, reassurance and general skin care focusing on avoiding skin dryness are required. Emollients, keratolytic agents, topical steroids, chemical or mechanical peels, vitamin D3 analogues, and topical or systemic retinoids may be used [8]. Various laser or light-based therapies targeting different components of the lesion have been tried with variable success rate [4, 5, 10, 11]. Despite the advantage of low cost, topical treatments have limited success rate, as they may not target all components of KP (e.g., hyperkeratosis, hyperpigmentation, and erythema). In addition, irritation from high-concentrated keratolytic agents can be intolerable to some patients. According to the histopathologic nature of KP, epidermal hyperkeratosis, hypergranulosis, and plugging of individual hair follicles are seen [3, 12]. Mild superficial perivascular lymphocytic inflammatory infiltrates can be found which reflex the erythematous component of some KP lesions. In Fitzpatrick skin types III–V (i.e., Asian skin), brown pigmentation is often seen [7]. In this study, the rationale for the use of fractional CO_2_ is to target the excess keratinous plug and the brown pigments.

In this study, we demonstrated that a single session of fractional CO_2_ laser treatment results in moderate to good improvement of KP lesions in some patients by global assessment. Although merely 30% of the patient had moderate to good improvement at the end of the study, to the patients' perception, all stated that the laser treatment improved the appearance of their lesions.

Taking particular elements of KP into account, keratotic papules and hyperpigmentation appear to respond better than erythematous components. Evidently, higher numbers of patients rated their keratotic lesions and hyperpigmentation as moderate or good response on both 4 weeks and12 weeks of follow-up. This result supports our hypothesis that fractional CO_2_ laser treatment could eradicate excess keratosis and pigmentation.

Regarding the laser parameter in this study, the pulse energy of 24–30 mJ with a spot density of 300 spots/cm^2^ was delivered into 2 passes. This setting was capable of causing maximum ablative depth of approximately 530–580 *μ*m and the width of coagulative zone is 243 *μ*m per dot, whereas the epidermal thickness of the shoulder and upper extremities is approximately 81–135 *μ*m [13, 14]. With this setting, we believe that certain parts of each keratotic papule would be destroyed. However, due to disparity in size and depth of keratotic papules, absolute ablative depth may be variable. Hence, some may be completely ablated and others may only be partially ablated.

There was marginal degree of improvement on KP lesions of control side (side B). This is probably due to the emollient effect from petrolatum ointment. As also demonstrated the mean improvement score of side B at the 12th week of follow-up was less than that of the 4th week of follow-up. Therefore, emollients are proven beneficial in KP when given continuously. Cessation of emollient may cause gradual recurrence. This same effect also occurred on side A, where the mean improvement score at the 4th week was better than the 12th week. For this reason, application of emollient should be advised to all patients, regardless of laser therapy.

Previous studies have evaluated the effectiveness of various lasers intended to attack specified targets such as 532 nm potassium titanyl phosphate laser and 595 nm pulsed dye laser aimed to reduce KP-associated erythema [4, 5, 10]. According to a study by Alcántara González et al., all 10 patients with keratosis rubra pilaris or keratosis pilaris atrophicans faciei achieved more than 75% improvement in erythema after 2–7 sessions of 595 nm pulsed dye laser [10]. In patients with pronounced pigmentary component of KP, 1064 nm Q-switched Nd:YAG laser may be used [6, 7]. Park et al. evaluated the efficacy of 1064 nm Q-switched Nd:YAG laser on pigmented KP, 41.7% of patients showed more than 50% improvement in dyspigmentation [6]. Recently, Saelim et al. successfully treated KP with long-pulsed 1064 nm Nd:YAG laser [9]. A significant improvement in global assessment, erythema, and keratotic papules was noted after 3 sessions of long-pulsed 1064 nm Nd:YAG laser at 4-week interval. The proposed mechanism of long-pulsed 1064 nm Nd:YAG laser was to reduce the size of the affected hair follicles. According to a study by Lee et al., the combination of 595 nm pulsed dye laser, long-pulsed 755 nm alexandrite laser, and microdermabrasion to target 3 components of KP resulted in marked improvement of KP in 51.7% of patients [8]. Therefore, the most suitable choice for the treatment of KP mainly depends on the pronounced and problematic component. Combination of different lasers or modalities may be most beneficial to patients with multiple elements of KP. However, lasers hold several limitations such as pain, stinging sensation, erythema, purpura, hyper- and hypopigmentation, and high cost [4–10]. In addition, recurrence of the lesions may occur after treatment cessation.

The limitations of this study are the small sample size and the short follow-up time of 3 months. Therefore, a conclusion cannot be drawn as to how long the laser effect would last and whether recurrence would occur. Moreover, we did not count the actual keratotic lesions and skin roughness was inaccessible through the evaluation by 2D photography. Finally, our study was performed in Asian subjects with Fitzpatrick skin types III–V; hence, this laser setting cannot be applied to all skin types.

In conclusion, we demonstrated that fractional CO_2_ laser can be used as an alternative treatment for KP in some patients, particularly in the presence of marked keratotic components. However, special caution should be given to patients with greater Fitzpatrick skin type, as pigmentary alteration can occur as a sequel. Further studies are needed to find the optimum parameter, appropriate frequency, and suitable treatment sessions of fractional CO_2_ laser for KP.

## Figures and Tables

**Table 1:** Global assessment by nontreating dermatologists.

Grading	4 weeks of follow-up, *n* = 20		12 weeks of follow-up, *n* = 20	
	Side A	Side B	Side A	Side B
0 (no improvement)	8	12	13	16
1 (1–25% improvement, minimal)	8	7	1	4
2 (26–50% improvement, moderate)	2	1	5	—
3 (51–75% improvement, good)	2	—	1	—
4 (>75% improvement, excellent)	—	—	—	—

Side A: fractional CO_2_ laser treated side.

Side B: control side.

**Table 2:** Assessment of keratotic papules by nontreating dermatologists.

Grading	4 weeks of follow-up, *n* = 20		12 weeks of follow-up, *n* = 20	
	Side A	Side B	Side A	Side B
0 (no improvement)	11	15	8	14
1 (1–25% improvement, minimal)	5	4	7	5
2 (26–50% improvement, moderate)	1	1	3	1
3 (51–75% improvement, good)	3	—	2	—
4 (>75% improvement, excellent)	—	—	—	—

Side A: fractional CO_2_ laser treated side.

Side B: control side.

**Table 3:** Assessment of pigmentation by nontreating dermatologists.

Grading	4 weeks of follow-up, *n* = 20		12 weeks of follow-up, *n* = 20	
	Side A	Side B	Side A	Side B
0 (no improvement)	13	14	13	16
1 (1–25% improvement, minimal)	2	6	1	4
2 (26–50% improvement, moderate)	5	—	5	—
3 (51–75% improvement, good)	—	—	1	—
4 (>75% improvement, excellent)	—	—	—	—

Side A: fractional CO_2_ laser treated side.

Side B: control side.

**Table 4:** Patiens' satisfaction at 12 weeks of follow-up.

Patients' grading	Side A, *n* = 20	Side B, *n* = 20
0 (unsatisfied)	—	5
1 (poor)	8	11
2 (fair)	5	4
3 (satisfied)	5	—
4 (extremely satisfied)	2	—

Side A: fractional CO_2_ laser treated side.

Side B: control side.
